# Identification and Characterization of Proteins Involved in Nuclear Organization Using *Drosophila* GFP Protein Trap Lines

**DOI:** 10.1371/journal.pone.0053091

**Published:** 2013-01-16

**Authors:** Margaret Rohrbaugh, Alyssia Clore, Julia Davis, Sharonta Johnson, Brian Jones, Keith Jones, Joanne Kim, Bramwel Kithuka, Krystal Lunsford, Joy Mitchell, Brian Mott, Edward Ramos, Maza R. Tchedou, Gilbert Acosta, Mark Araujo, Stuart Cushing, Gabriel Duffy, Felicia Graves, Kyler Griffin, B. V. Gurudatta, Deaundra Jackson, Denis Jaimes, Kendall Jamison, Khali Jones, Dhaujee Kelley, Marquita Kilgore, Derica Laramore, Thuy Le, Bakhtawar Mazhar, Muhammad M. Mazhar, Britney McCrary, Teanndras Miller, Celethia Moreland, Alex Mullins, Elyas Munye, Sheila Okoorie, Elisha Pittman, Nikkita Roberts, De’Warren Rose, Alex Rowland, Anwar Shagarabi, Jamela Smith, Tayler Stallworth, Nicole Stroud, Elizabeth Sung, Kai Sung, Naomi Takenaka, Eduardo Torre, Jarvis Veira, Kim Vu, William Wagstaff, Ashley M. Wood, Karen Wu, Jingping Yang, Victor G. Corces

**Affiliations:** 1 Department of Biology, Emory University, Atlanta, Georgia, United States of America; 2 South Atlanta High School of Health and Medical Science, Atlanta, Georgia, United States of America; 3 Carver School of Health Science and Research, Atlanta, Georgia, United States of America; 4 Baltimore Polytechnic Institute, Baltimore, Maryland, United States of America; 5 Paul Laurence Dunbar High School, Baltimore, Maryland, United States of America; 6 North Atlanta High School, Atlanta, Georgia, United States of America; 7 Cross Keys High School, Atlanta, Georgia, United States of America; 8 Frederick Douglass High School, Atlanta, Georgia, United States of America; Bellvitge Biomedical Research Institute (IDIBELL), Spain

## Abstract

**Background:**

Strains from a collection of *Drosophila* GFP protein trap lines express GFP in the normal tissues where the endogenous protein is present. This collection can be used to screen for proteins distributed in the nucleus in a non-uniform pattern.

**Methodology/Principal Findings:**

We analyzed four lines that show peripheral or punctate nuclear staining. One of these lines affects an uncharacterized gene named *CG11138*. The CG11138 protein shows a punctate distribution in the nuclear periphery similar to that of *Drosophila* insulator proteins but does not co-localize with known insulators. Interestingly, mutations in Lamin proteins result in alterations in CG11138 localization, suggesting that this protein may be a novel component of the nuclear lamina. A second line affects the *Decondensation factor 31* (*Df31*) gene, which encodes a protein with a unique nuclear distribution that appears to segment the nucleus into four different compartments. The X-chromosome of males is confined to one of these compartments. We also find that *Drosophila* Nucleoplasmin (dNlp) is present in regions of active transcription. Heat shock leads to loss of dNlp from previously transcribed regions of polytene chromosome without redistribution to the heat shock genes. Analysis of Stonewall (Stwl), a protein previously found to be necessary for the maintenance of germline stem cells, shows that Stwl is present in a punctate pattern in the nucleus that partially overlaps with that of known insulator proteins. Finally we show that Stwl, dNlp, and Df31 form part of a highly interactive network. The properties of other components of this network may help understand the role of these proteins in nuclear biology.

**Conclusions/Significance:**

These results establish screening of GFP protein trap alleles as a strategy to identify factors with novel cellular functions. Information gained from the analysis of CG11138 Stwl, dNlp, and Df31 sets the stage for future studies of these proteins.

## Introduction

The three-dimensional organization of the chromatin fiber in the nucleus is important for the spatial and temporal regulation of transcription during development [1], [2]. One class of proteins that appears to be involved in the establishment and/or maintenance of this organization is insulator proteins. Previous studies have shown that *Drosophila* insulator proteins are present at several thousand sites throughout the genome where they may play a variety of functions [3]–[6]. Insulators mediate inter- and intra-chromosomal interactions between different sequences in the genome and their effect on gene expression depends on the nature of the sequences brought together [2]. These interactions can now be detected by 3C and related techniques but one of the first clues suggesting that individual insulator sites co-localize in the nucleus came from the use of immunofluorescence microscopy to observe the distribution of these proteins in cell nuclei. Results from these experiments indicated that insulator proteins co-localize to distinct foci named insulator bodies located in the nuclei of diploid cells [7].

The use of screens based on yeast two hybrid or classical genetics has allowed the identification of a number of proteins that interact with insulator DNA sequences or other insulator components in *Drosophila* but additional factors may be required to explain all the properties of these sequences [4], [8]–[10]. One approach that has not been previously explored is the use of microscopy-based screens to identify proteins located in the nucleus in a pattern similar to that of insulator bodies. Such approach may also afford the identification of additional proteins involved in other aspects of nuclear biology based on their subnuclear distribution. For example, Polycomb-Group (PcG) proteins are also present in a punctate pattern forming Pc bodies where loci silenced by H3K27me3 and PcG proteins come together in the nucleus [11]. Other nuclear compartments where various proteins are present in a distinct pattern include transcription factories, the nucleolus, PML bodies, Cajal bodies and Nuclear Speckles and Paraspeckles [12]. Therefore, it may be possible to identify protein components present at these different nuclear structures by using microscopy-based screens to visualize proteins present in an anisotropic nuclear distribution pattern.

We have used immunofluorescence microscopy of live tissues to screen a collection of GFP-tagged protein-trap *Drosophila* alleles with the goal of identifying proteins with interesting nuclear localization patterns [13]–[15]. Once a protein was identified, further analysis was carried out to elucidate its role in chromatin organization and nuclear biology. In addition to its importance in the characterization of proteins with a role in specific nuclear processes, this approach meets a number of important criteria that facilitate its use for educational purposes and was used to allow high school and college students to experience the process of research in biology. Here we describe the results of this microscopy-based screen for proteins present in a non-diffuse pattern in the nucleus of *Drosophila* cells. We focus our analysis on four of these proteins, *Drosophila* Nucleoplasmin (dNlp), Decondensation factor 31 (Df31), Stonewall (Stwl) and CG11138, that show a punctate distribution similar to that of insulator or Pc bodies. The results give a glimpse into the function of these proteins in nuclear biology and set the stage for future investigation of the role of these proteins in chromatin structure and transcription.

## Results

### Screen of a GFP Protein-Trap Collection Allows the Identification of Proteins with Distinct Nuclear Distributions

In order to identify proteins present in a distinct pattern in the nucleus, we dissected third instar larvae from strains expressing GFP fused to different *Drosophila* proteins [13]–[15]. Imaginal discs and salivary glands were isolated and the GFP fluorescence signal was observed using live-imaging fluorescence microscopy. We screened a total of 606 lines, of which 486 were GFP-positive and 141 had nuclear GFP staining. Of these, 83 were insertions in previously characterized genes, 32 correspond to uncharacterized genes and 26 were not mapped. Seventy six lines were selected and further analyzed in detail to determine the distribution pattern of the GFP fluorescence in the nucleus. Only those lines in which the pattern of GFP localization shows a non-uniform subnuclear distribution were selected for additional study. Figure S1 shows examples of nuclear and non-nuclear GFP auto-fluorescence in diploid cells and salivary gland cells containing polytene chromosomes. Lines with insertions of GFP in genes previously well characterized were discarded and only those fly lines with inserts in novel or not well studied proteins were kept. In total, about 13% of the lines screened were classified as of potential interest based on the GFP distribution pattern. Four of these were studied further, including the uncharacterized protein CG11138, Decondensation factor 31 (Df31), Stonewall (Stwl), and *Drosophila* Nucleoplasmin (dNlp).

### CG11138 Encodes a Putative Ubiquitin E3 Ligase Present in the Nuclear Lamina

**Figure 1:**
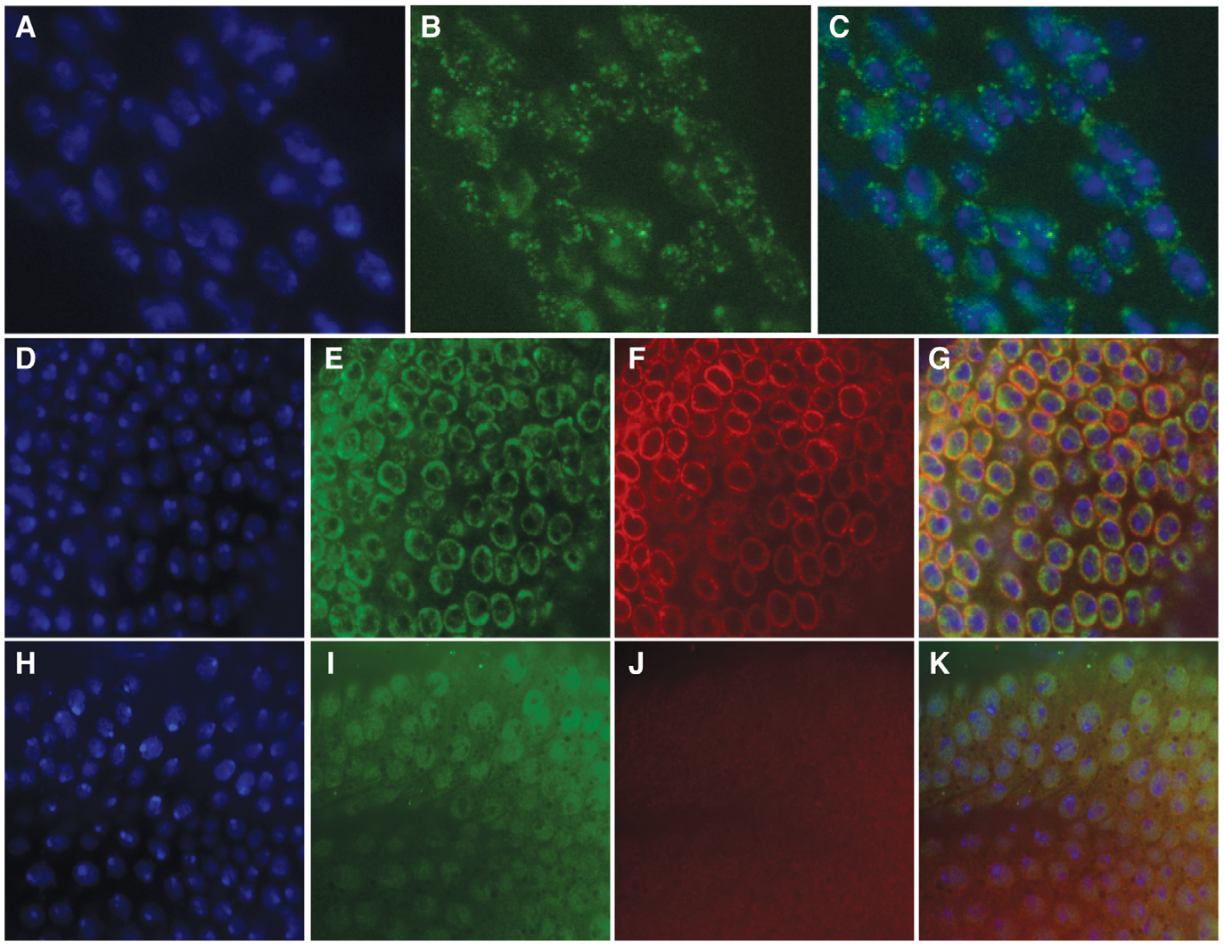
CG11138 is localized at the nuclear periphery and this localization is disrupted in the absence of Lamin C. Panels (A–C) depict diploid cells from third instar larvae imaginal tissue of the CG11138 protein-trap allele labeled with DAPI (A), α-GFP (B) and merged (C) indicating that CG11138-GFP is localized in punctate bodies mainly around the nuclear periphery. Panels (D–G) show diploid cells from OR third instar larvae imaginal tissue labeled with DAPI (D), α-CG11138 (E), α-Lamin C to define the nuclear periphery (F) and the merged image (G). Panels (H–K) show DAPI (H), α-CG11138 (I), α-LaminC (J) and merged (K) images of diploid cells from third instar larvae imaginal tissue of *lamC^SZ18^/lamC^SZ18^* mutant flies. Loss of Lamin C disrupts the localization of CG11138.

**Figure 2:**
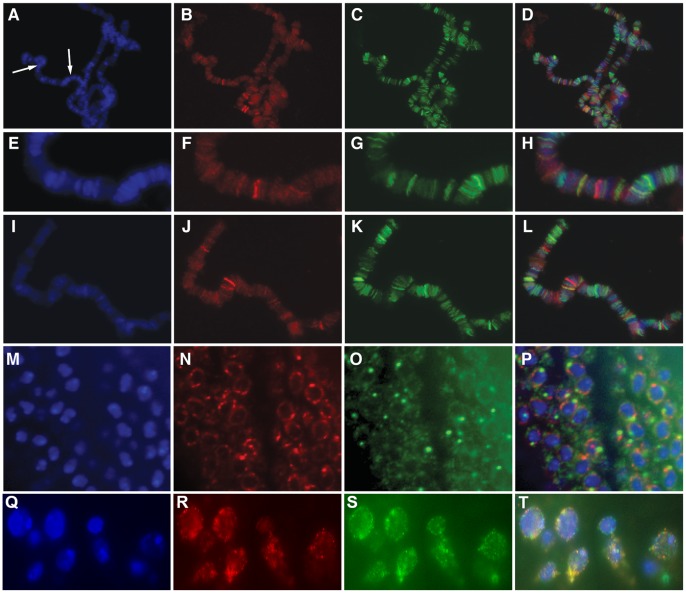
CG11138 does not significantly colocalize with insulator proteins on polytene chromosomes or in diploid cells. Panels (A–D) show labeling of polytene chromosomes with DAPI (A), α-CG11138 (B), α-Mod(mdg4)2.2 (C) as well as the merged image (D). Panels E–H show an enlarged portion of the chromosome displayed in panels A–D in the region defined by the white arrows. Panels I–L show a second region of a different chromosome labeled with antibodies to CG11138 and Mod(mdg4)2.2, further underscoring the limited amount of colocalization between these two proteins. Panels M-O show diploid cells from OR third instar imaginal tissue labeled with DAPI (M), α- CG11138 (N) and α-CP190 (O). Panel P shows the merged image indicating that CP190 and CG11138 do not overlap in diploid cells and specifically the bodies formed by each protein appear mutually exclusive. Panels Q–T show diploid cells from OR third instar imaginal tissue labeled with DAPI (Q), α- CG11138 (R) and α-dTopors (S). Panel T shows the merged image indicating that dTopors and CG11138 do not significantly overlap in diploid cells and specifically the bodies formed by each protein appear mutually exclusive with some exceptions were they seem to overlap.

The protein trap line CA06844 contains an insertion of GFP in the *CG11138* gene. The GFP-tagged CG11138 protein is distributed in punctate bodies present throughout the nucleus (Figure 1A–1C). This pattern of nuclear localization is similar to that seen for insulator proteins [7], [9]. CG11138 is a novel protein with no identified molecular function, an ideal candidate for further study. CG11138 shares similarities with mammalian Interferon regulatory factor 2- binding proteins1 and 2 (IRF2-BP1 and 2) that act as transcriptional co repressors with IRF2 (Childs and Goodbourn 2003). The similarity between the two proteins is centered around a C3HC4 Ring-type zinc finger domain that is characteristic of E3 ubiquitin ligases, suggesting that, as is the case for IRF2-BP1 [16], CG11138 may be involved in ubiquitination of other proteins. The *CG11138* gene encodes three predicted protein isoforms of different molecular weights, CG11138-PB (24.5 kDa), CG11138-PC (60.0 kDa) and CG11138-PD (61.6 kDa). In order to study the role of these proteins, we prepared antibodies against a region common to all three isoforms. Western analysis using these antibodies suggests that the different isoforms are differentially expressed during *Drosophila* development, with the smallest isoform being absent in third instar larvae (Figure S2). A major band is seen at 65 kDa, which is close to the predicted size of isoforms CG11138-PC and CG11138-PD [17]. In addition, two larger proteins of approximately 100 kDa and 150 kDa also appear to be encoded by *CG11138*, as they shift in size when the GFP-tagged protein is identified from third instar imaginal tissue of the CG11138 protein-trap allele (Figure S2 lanes 4–6). The larger than predicted size of these proteins may be due to covalent modification. Immunostaining of imaginal discs from third instar larvae shows that the nuclear localization of CG11138 is very similar to that seen using antibodies directed against GFP in the protein-trap allele, indicating that screening for GFP fluorescence in cell nuclei can be a viable way to identify novel nuclear proteins with interesting distribution patterns (Figure 1B and 1E). CG11138 is predominantly localized to the nuclear periphery with only a few punctate bodies found within the nucleus (Figure 1D–1G). Colocalization studies suggest that CG11138 overlaps with both Lamin C (Figure 1G) and Lamin Dm0 at the nuclear periphery but appears to be contained within Lamin boundaries (data not shown but nearly identical to data shown for Lamin C). It is interesting to note that this subnuclear localization, as well as the presence at the borders between bands and interbands, is similar to that observed for dTopors, another E3 ubiquitin ligase involved in the regulation of the Su(Hw) insulator [8]. This distribution pattern is disrupted by mutations in both *lamin C* (Figure 1H–2K) and *lamin Dm0* (similar to that shown for *lamin C*). The disruption of CG11138 distribution in the nucleus may be a consequence of its direct interaction with Lamin proteins or a secondary consequence from the disruption of the nuclear lamina and corresponding alteration in nuclear architecture that occurs when Lamins are mutated.

The CG11138 protein is present at the band/interband border of polytene chromosomes (Figure 2A–2L). This pattern closely resembles that of other *Drosophila* insulator proteins [7], [9], [18]. However, immunofluorescence studies indicate a lack of strong co-localization between CG11138 and insulator proteins on polytene chromosomes. Figure 2 depicts polytene chromosomes labeled with antibodies directed against both CG11138 and Mod(mdg4)2.2, a component of the Su(Hw) insulator complex. Both proteins are present in multiple regions but the amount of overlap is only about 20%. Interestingly, both proteins are found at band/interband borders and thus are often found adjacent to each other but not overlapping as can be seen in the enlarged polytene chromosome regions (Figure 2H and 2L). Furthermore, although CG11138 is present at punctate bodies around the nuclear periphery of diploid cells, it does not overlap with insulator bodies formed by known insulator proteins (Figure 2M–2P). In fact, costaining of CG1138 with Centrosomal Protein 190 (CP190), a protein found associates with several classes of *Drosophila* insulator complexes, suggests that the distributions of these two proteins seem to be mutually exclusive. Given the similarities between CG11138 and the E3 ubiquitin ligase dTopors, which associates with the Su(Hw) insulator [8], we examined their nuclear distribution by immunofluorescence microscopy using imaginal disc cells. The results suggest that the two proteins are present at foci immediately adjacent to each other but with little overlap (Figure 2Q–2T).

### Decondensation Factor 31

A second protein identified in the GFP trap screen is Decondensation factor 31 (Df31). Previous studies suggest that Df31 is a highly disordered protein with the potential for interaction with different binding partners [19] and is able to de-condense *Xenopus* sperm DNA *in vitro* [20]. Df31 is expressed at every stage of the *Drosophila* life cycle and appears to be closely associated with DNA [20], [21]. Injection of Df31 antisense RNA into *Drosophila* embryos results in an abnormal number of nuclei and clumping or disorganization of the chromatin and eventual lethality [21]. *In vitro* studies suggest that Df31 can associate with histone H3 tails, can bind oligonucleosomes and can facilitate inter-strand bridging between chromatin substrates [22]. More recently, Df31 has been shown to bind to snoRNAs and tether this protein-RNA network to active chromatin [23]. These results suggest that Df31 may play a role in either the assembly or maintenance of chromatin structure. However, since the majority of this work was done *in vitro*, the *in vivo* function of Df31 in the nucleus has yet to be clearly defined.

**Figure 3:**
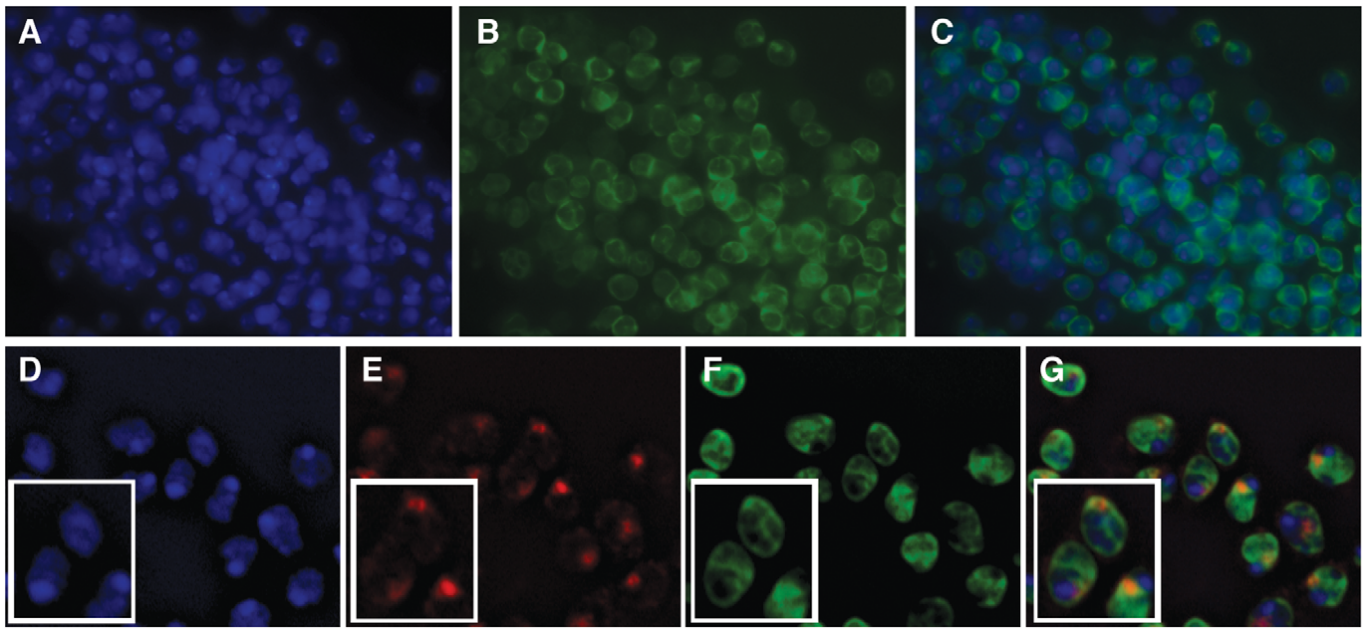
Df31 localization divides diploid cell nuclei into four quadrants. Panels A–C show Df31-GFP localization in diploid cells from Df31 protein-trap third instar larvae imaginal tissue; DAPI (A), α-GFP (B), and merged (C). Panels D-G depict Df31-GFP and MSL1 costaining in diploid cells from male Df31 protein-trap third instar larvae imaginal tissue; DAPI (D), α-MSL1 (E), α-Df31 (F) and merged (G). The insert in panels D–G show a close-up of individual cells to more clearly depict the segregation of MSL1 to one Df31 quadrant.

Analysis of GFP fluorescence of protein trap alleles of Df31 suggests that this protein is distributed around the polytene chromosomes of salivary gland nuclei rather than at the chromosomes; its localization in diploid cells is rather diffuse throughout the nucleus but there are distinct regions of localization (Figure S1C–S1D). Further, immunostaining of the protein trap alleles with antibodies to GFP shows a very unique localization in diploid cells, with GFP-Df31 forming a cross-like pattern that appears to divide the nucleus into four quadrants (Figure 3A–3C). It is possible that the pattern seen in these alleles is a consequence of elevated amounts of GFP-Df31 protein within the nucleus of cells from strains carrying the GFP protein trap. To address this question, we prepared antibodies against Df31 and carried out immunohistochemical analyses in cells from imaginal discs of third instar larvae. Df31 shows a similar cross-like pattern that is less clearly defined, but still present, in wild type flies. In these cells, Df31 also appears to localize to the nuclear periphery with faint lines observed inside the nucleus (Figure 3G).

The distribution pattern of Df31 is of particular interest as *Drosophila* contains four chromosome pairs that occupy four chromosome territories in diploid cells [24]–[26]. It is therefore possible that Df31 may play a role in establishing or maintaining this organization. To address this possibility, we co-stained diploid nuclei from males of Df31 protein-trap alleles with antibodies against GFP and MSL-1, a protein known to specifically bind the male X chromosome [27], [28]. Figures 3D–3G depict results from these experiments using confocal microscopy followed by 3D reconstruction of the nuclear space. The results indicate that MSL-1 is confined within one Df31 quadrant. This suggests that each quadrant delimited by Df31 may represent a chromosome territory. These data do not shed light, however, on whether Df31 is required to establish or maintain chromosome territories. Based on the high amount of Df31-GFP fusion protein found surrounding polytene chromosomes in the protein-trap allele and its predicted ability to have several binding partners [19], it is possible that Df31 may simply be occupying regions surrounding the chromosomes and thus it appears to delineate territories. This hypothesis agrees well with the finding that Df31 forms a network by interacting with snoRNAs and chromatin, suggesting that this protein-RNA network may be part of the nuclear matrix. Disruption of Df31 expression may help to dissect its possible role in establishing or maintaining chromosome territories and more clearly define its role in nuclear biology.

### Drosophila Nucleoplasmin is Present in Interbands of Polytene Chromosomes and is Released after Heat Shock

**Figure 4:**
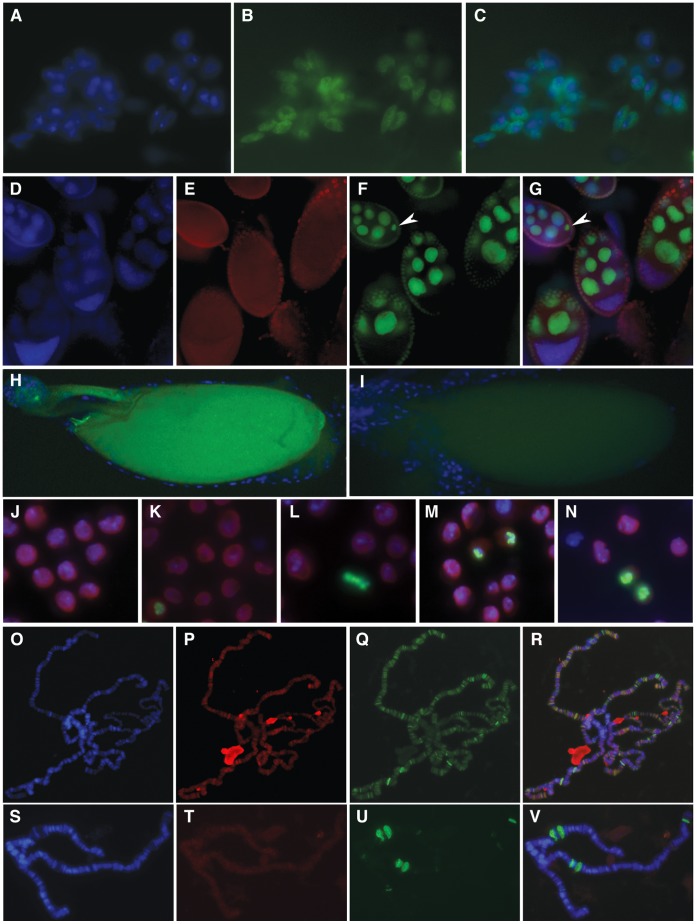
Distribution of dNLP in various cell types. (A–C) dNLP-GFP flourescence in diploid cells from third instar larvae imaginal tissue from a dNLP protein-trap allele; DAPI (A), dNLP-GFP (B) and merged (C). Panels (D–G) depict dNLP localization in both somatic and germline cell nuclei including the oocyte nucleus (arrowhead in F and G); (D) DAPI, (E) α- Lamin Dm0, (F) α-dNLP and (G) merged. Panels H and I show dNLP-GFP fluorescence in an egg from a dNLP protein-trap allele (H) as compared to a wild type egg (I) indicating that dNLP-GFP is being dumped into the developing egg of the protein-trap allele. Panels J-N show Kc cells labeled with DAPI- blue, α-dNLP- red and α-H3S10ph-green in interphase (J), prophase (K), metaphase (L), anaphase (M) and telophase (N); the results suggest that dNLP is not associated with condensed chromosomes during metaphase. Panels (O–V). (O–R) show polytene chromosomes from wild type third instar larvae prior to heat shock while (S–V) show chromosomes from larvae subjected to a 20 min heat shock at 37°C. dNLP is broadly present in interbands and frequently colocalizes with RNA Pol II phosphorylated at Ser5 on the non-heats hocked polytene chromosomes. dNLP seems to become more diffuse and dissociate from the DNA following heat shock. The distribution of dNLP is particularly weak at the heat shock puffs, which are the only sites of transcription following temperature elevation (S–V). (O and S)-DAPI, (P and T)-α-dNLP, (Q and U)-α-PolII^ser5^ and (R and V)-merged.

A third protein identified in the protein-trap screen is *Drosophila* Nucleoplasmin (dNlp). Published work suggests that dNlp can bind core histones, facilitating nucleosome assembly, and is capable of decondensing *Xenopus* sperm *in vitro* [29]. Protein trap lines expressing a GFP-dNlp fusion protein show nuclear fluorescence (Figure 4A–4C). To determine whether dNlp could play a similar role in *Drosophila*, we analyzed its distribution in the female ovary. dNlp is highly expressed in nurse cells during oogenesis and is also present in the egg (Figure 4D–4I). It is therefore possible that dNlp may be deposited in the egg to assist in sperm DNA decondensation following fertilization. In order to investigate other possible roles of dNlp in chromosome decondensation, we examined its subcellular distribution during different stages of the cell cycle (Figure 4J–4N). dNlp is not present on mitotic chromosomes and, therefore, an involvement in their decondensation at the end of mitosis is unlikely (Figure 4L–4N).

We have used dNlp antibodies to analyze the possible role of this protein in transcription by affecting chromatin condensation. Results from these studies suggest that dNlp binds specifically within the interbands of polytene chromosomes where transcriptionally active genes are located, suggesting that it may play a role in transcription (Figure 4O–4R). Since dNlp has been previously shown to function as a histone chaperone, it is possible that its role in transcription takes place at the level of the nucleosome. To further assess the function of dNlp in the transcription process, the heat-shock response was used as an experimental paradigm. During heat-shock, transcription of all genes is repressed while a small number of heat shock genes become transcribed. Following a 20 min heat-shock, dNlp disappears from the pre-existing sites on the polytene chromosomes but shows no strong accumulation at the heat-shock genes. Interestingly, dNlp appears to concentrate in the nucleolus before heat shock but it is absent after temperature elevation. As Figure 4 suggests, the small amount of dNlp that is detected on the chromosomes following heat shock has become diffuse and no longer binds to distinct regions, particularly at the sites of the heat shock genes, which appear to have no dNlp. It is possible that dNlp may be needed only for the very initial stages of transcription and thus cannot be detected when the heat shock response genes have been activated for a period of time. Another possibility is that Poly(ADP-ribosyl)ation disrupts nucleosomes to facilitates transcription of heat shock genes, and that dNlp may not be required as a chaperone by these genes. It is interesting to note that dNlp is present in the nucleolus at normal temperature but is absent after heat shock.

### Stonewall is Distributed in a Punctate Pattern in Germline and Somatic Cells

Stonewall (Stwl) has been shown to be essential to maintain germline stem cells in the adult ovary [30], [31]. Previous work has also shown that Stwl is a dominant suppressor of position effect variegation [32]. Furthermore, using microarray analysis, it has been found that loss of Stwl can alter the expression of several hundred genes [33]. Stwl may act as part of a chromatin remodeling complex but the nature of other proteins in the complex has not been defined [32], [33]. Previous studies of Stwl suggest that this protein is not highly expressed in cells outside of the ovary.

**Figure 5:**
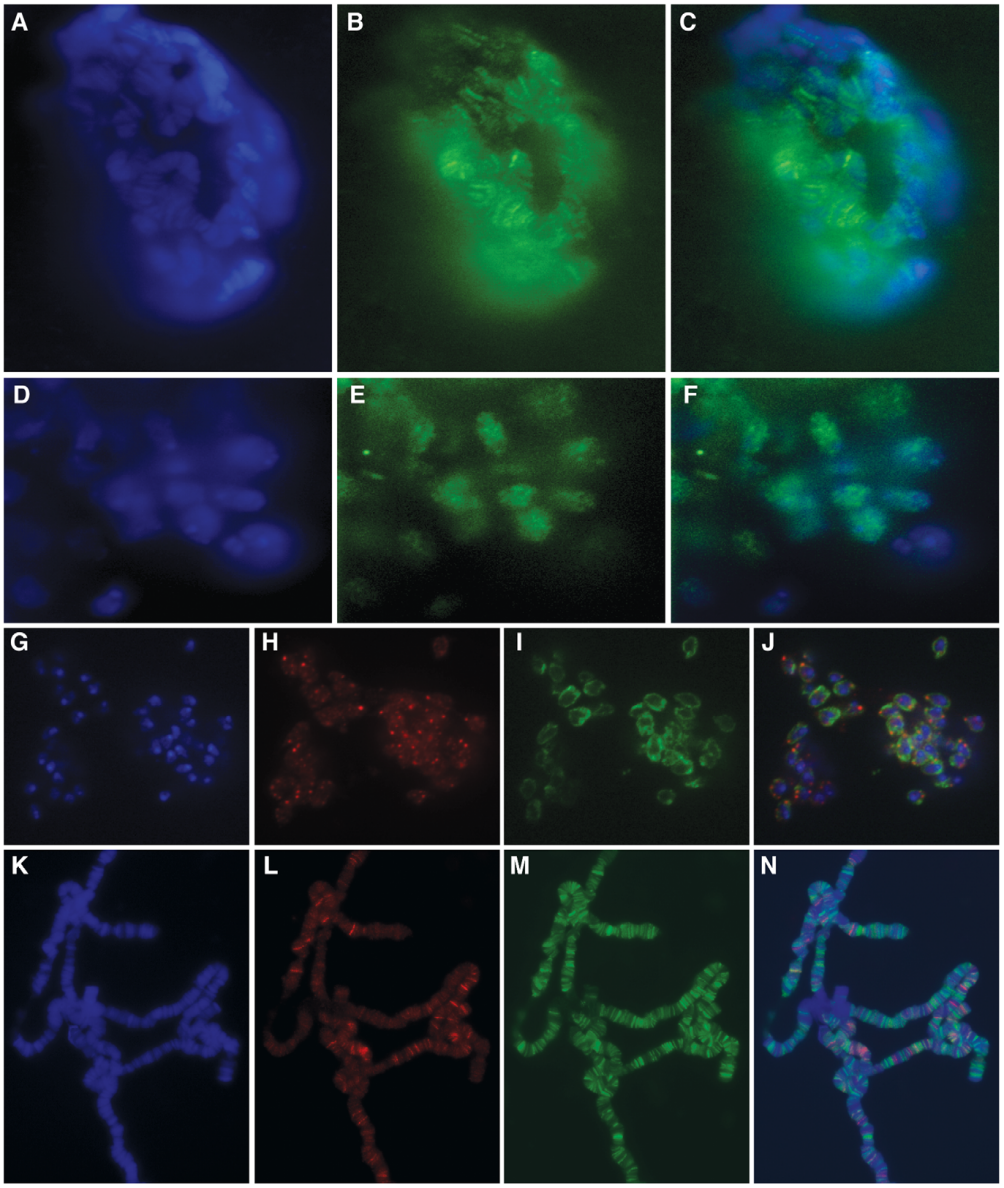
Stwl is present in polytene chromosomes and in distinct foci in the nucleus of diploid cells. Panels (A–F) show GFP fluorescence for Stwl-GFP in polytene chromosomes (A–C) and in diploid cells (D–F). (A and D) show DNA labeled in blue by DAPI. Stwl-GFP is shown in green in panels (B and E) while panels (C and F) show the merged images. Stwl localization appears as dots around the periphery of diploid cells; DAPI (G), α-Stwl (H), α-Lamin Dm0 (I) and merged (J). Panels (K–N) show polytene chromosomes labeled with Stwl and CP190 antibodies indicating little overlap between the two proteins, DAPI (K), α-Stwl (L), α-CP190 (M) and merged (N).

**Figure 6:**
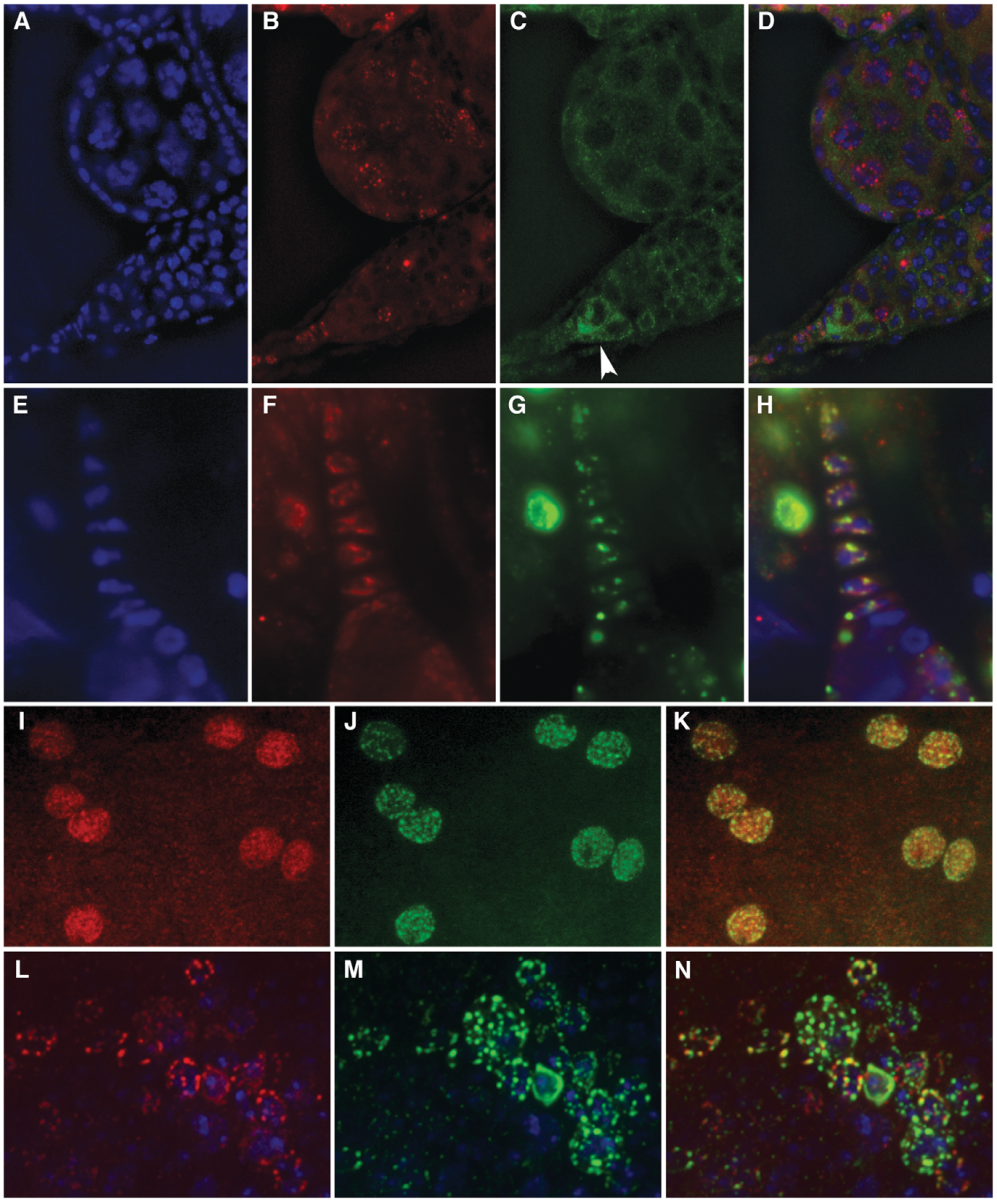
Stwl expression in germline stem and differentiated cells. Panels (A–D) show the germarium of a single ovariole in the ovary (lower portion of panels) as well as an early stage egg chamber (upper portion of panel). In (A) nuclei are labeled with DAPI, (B) shows α-Stwl staining, (C) outlines the germline stem cells (arrowhead) using α-Vasa and (D) shows the merged image. Stwl colocalizes with CP190 in ovarian somatic cells. Panels (E–N) show Stwl and CP190 colocalization in terminal filament cells (E–H), follicle cells (I–K), and imaginal disc cells (L–N); DAPI is blue (E), α- Stwl is red (F, I and L), α-CP190 is green (G, J and M) and in the merged panels yellow regions show colocalization (H, K and N).

Analysis of Stwl-GFP protein trap lines shows that this protein is present at many sites on polytene chromosomes, suggesting that it may bind DNA at multiple genomic locations in somatic cells (Figure 5A–5C). In addition, Stwl is distributed in a nuclear punctate pattern in diploid cells (Figure 5E). Analyses of Stwl distribution in adult ovaries using both the protein-trap alleles and wild type flies indicate that this protein is present in a diffuse localization pattern in germline stem cell nuclei, which quickly becomes more punctate during cystoblast development and in the nurse cells of the developing egg chamber. Figure 6 shows the germarium of a wild type adult female ovary in which Stwl and Vasa proteins have been labeled by immunohistochemistry. Two large germline stem cells can be clearly seen at the tip of the germarium directly below the terminal filament cells. Stwl is diffusely distributed in these cells but appears to localize preferentially to the nuclear periphery. In adjacent cells of the developing cystoblast, however, Stwl becomes localized into distinct foci around the periphery of the nucleus (Figure 6A–D). Stwl does not appear to be expressed in all cells of the developing cystoblast but it is present in the nurse cells of later stage egg chambers. In addition, Stwl can be found in distinct foci of the terminal filament cells as well as follicle cells surrounding the germarium and egg chambers (Figure 6E–K). These properties are similar to those observed for insulator proteins, which bind polytene chromosomes at many locations but these sites coalesce into a few foci in diploid nuclei. It is then possible that Stwl may play a role similar to that of insulator proteins by mediating interactions between distant sequences in the genome. To test whether Stwl may be a component of other *Drosophila* insulators, colocalization studies were done in female ovaries using antibodies to Stwl and CP190. The results from these experiments suggest that Stwl and CP190 appear to colocalize both in the terminal filament cells, which are known to regulate germline stem cells [34], and in the follicle cells surrounding the egg chambers (Figure 6H and 6K). The same is true in imaginal disc cells (Figure 6L–6N). It should be noted that there is not complete overlap between these two proteins, as distinct foci can be seen for each protein alone.

Further analyses confirm that Stwl is strongly expressed in diploid cells of third instar imaginal tissue as well as binding to several regions on polytene chromosomes (Figure 5G–N). It is interesting to note that there does not appear to be a significant overlap between Stwl and CP190 on polytene chromosomes despite the fact that these two proteins partially colocalize in somatic cells of the ovary (Figures 5N, 6H and 6L). Similar to what was seen for CG11138, Stwl, which binds to interbands often close to band/interband borders, is often adjacent to CP190 but does not appear to overlap. It is possible that although these two proteins may bind distinct regions of DNA, these regions may come together by looping of the intervening sequences. Stwl is also localized in distinct foci mainly around the periphery of the nucleus in diploid cells as can be seen by the colocalization with Lamin Dm0 (Figure 5J). These results suggest that Stwl shares some properties with known insulator proteins but additional studies are necessary to determine whether Stwl has similar functional characteristics.

### Stwl, Df31, and dNlp are Part of a Large Protein-protein Interaction Network

**Figure 7:**
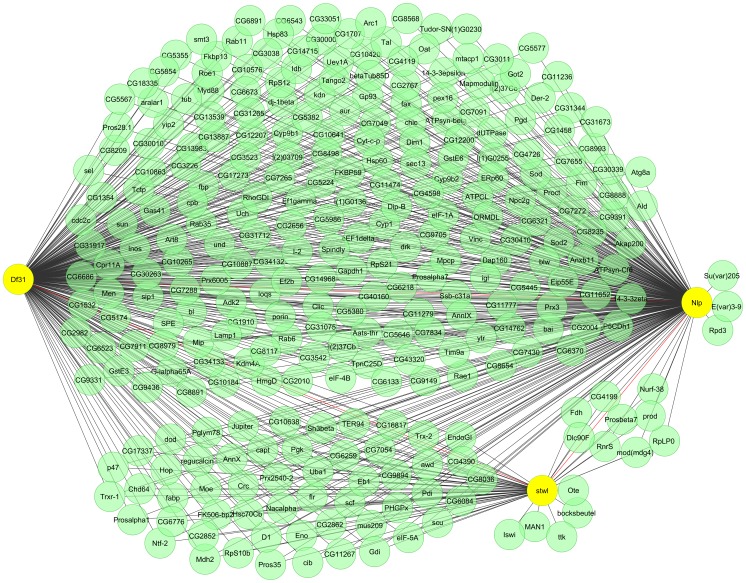
dNlp, Stwl and Df31 form part of a protein interaction network. A matrix of *Drosophila* interacting proteins was imported into Cytoscape and a child network was created by selecting the dNlp, Stwl and Df31 nodes plus all adjacent edges. For easier visualization, single nodes were deleted with the exception of those shown, which correspond to proteins of special interest. All other interactions are shown.

Artavanis-Tsakonas and collaborators have recently published results from an extensive study of protein-protein interactions in *Drosophila* cells [35]. The interaction partners of more than 5,000 *Drosophila* proteins were identified by co-affinity purification followed by mass spectrometry analysis of epitope-tagged proteins. Since Stwl and dNlp are present in a punctate pattern in the cell and the former partially co-localizes with insulator bodies, we used the published *Drosophila* protein interactome to examine the possibility that these proteins interact and perhaps gain information on the nature of other interaction partners that could shed light on the role of these proteins in nuclear biology. We could not examine possible interactions of CG11138 because this protein was not among those analyzed in the global interactome study. The results of this analysis indicate that Stwl, Df31 and dNlp interact with each other and form part of a large network of interacting proteins (Figure 7). The three nodes of the network, Df31, Stwl and dNlp, interact with a common subset of proteins (Figure 7). In addition, Df31 and Nlp share a different large set of common partners that do not interact with Stwl. It is important to note that Stwl interacts with three known components of the nuclear lamina, Otefin, Bocksbeutel and Man1. In addition, dNlp interacts with three proteins involved in heterochromatin silencing, namely Su(var)205/HP1, E(var)3-9 and Rpd3. Interestingly, Mod(mdg4) is a component of a sub-network shared between Stwl and dNlp. This observation may explain the partial overlap between the punctate nuclear localization pattern of Stwl and CP190.

## Discussion

An important question in current biology is whether the genetic material is arranged in a specific pattern in the nucleus of eukaryotic cells and whether this arrangement is a consequence of genome function, whether the structure elicits specific functional outcomes, or both. An essential step in addressing this question is the identification of factors that contribute to the establishment of nuclear organization. We have previously identified protein components of insulator complexes using classical genetics or the yeast two hybrid system. Examination of the distribution of GFP fused with various proteins in live tissues of *Drosophila* protein-trap strains affords an alternative strategy. By screening for proteins present in the nucleus in various distribution patterns one may be able to identify components of various nuclear compartments that contribute to the three-dimensional organization of the chromatin fiber. Results presented here underscore the feasibility of this approach.

We have identified four different proteins that are present in a punctate or peripheral pattern in the nucleus. These four proteins, CG11138, Df31, dNlp and Stwl were either uncharacterized or studied as part of specific nuclear processes. The analyses of these proteins presented here advance our understanding of their function. For example, we show that the previously uncharacterized CG11138 protein is present in the nuclear periphery and overlaps with the nuclear lamina. Its distribution is dramatically disrupted in cells mutant for either of the two Lamin proteins present in *Drosophila*, suggesting that CG11138 may be an integral component of this structure. Understanding its precise role will require further studies, but given its homology to ubiquitin E3 ligases it is likely that CG11138 is involved in ubiquitination of other components of the nuclear lamina or of heterochromatin proteins present at the nuclear periphery. Similarly, the characterization of the distribution pattern of Df31 may offer important insights into the function of this protein. Df31 was initially characterized as a decondensation factor of sperm DNA. Nevertheless, Df31 appears to be also present in somatic cells where it is enriched in the inter-chromatin space. Although intriguing, the results do not unequivocally indicate that Df31 is involved in subdividing the nucleus into different territories, since it is possible that the cell has an abundance of this protein and that Df31 passively occupies the inter-chromosome nuclear space. Alternative, and in line with recent results, Df31 may form a network by interacting with snoRNAs and chromatin [23], suggesting that this protein-RNA network may be part of a nuclear matrix.

Nucleoplasmin and nucleophosmin are histone chaperones first characterized for their ability to decondense sperm chromatin [36]. Nucleoplasmin is present in large amounts in frog oocytes and can exchange sperm nuclear basic proteins to histones H2A and H2B during fertilization [37]. Interestingly, Nucleoplasmin is also involved in chromatin condensation during apoptosis [38]. Here we show that dNlp has a broad role in chromatin function, since it is present in the interbands of polytene chromosomes. Interbands contain de-condensed, actively transcribed chromatin, suggesting a role for dNlp in the transcription process. This is supported by the observation that dNlp does not bind to polytene chromosomes after heat shock in regions where transcription has been turned off. Surprisingly, dNlp is not present at the newly induced heat shock genes. Since these genes are rapidly induced after temperature elevation, it is possible that dNlp is not required for their transcription.

The last protein we chose to analyze is Stwl, which was initially characterized as a germline stem cell maintenance factor in *Drosophila*. Our results suggest a more general role for this protein in somatic cells given its broad distribution in polytene chromosomes. Interestingly, the nuclear distribution of Stwl changes dramatically when the female germline stems cells differentiate from a diffuse to a punctate pattern. If we interpret the Stwl bodies as sites in the nucleus where multiple Stwl-bound DNA sites come together, this change in Stwl distribution could represent a rearrangement of the three-dimensional architecture of the chromatin fiber as cells differentiate. The specific role of Stwl in nuclear biology is unclear at the moment but some insights may be gained by analyzing the nature of the proteins with which it interacts based on results from the *Drosophila* protein interactome. In addition to interacting with Df31, dNlp and Mod(mdg4), Stwl also associates with several proteins that have been extensively studied in the context of the role of chromatin in transcription. This includes the transcription factor tramtrack; Otefin, Boksbeutel and Man1, two LEM-domain proteins present in the nuclear lamina; the Iswi, and Nurf38 components of chromatin remodeling complexes; and Prod, a protein required for mitotic chromosome condensation. These results, together with findings showing their subnuclear localization, suggest that Df31, dNlp and Stwl may participate in gene expression processes that require the localization of the chromatin fiber to specific nuclear compartments. Although the understanding of the details of these processes will require further studies, these findings pave the way for future work in the field.

## Materials and Methods

### Ethics Statement

Antibodies used in this study were prepared by Pocono Rabbit Farm & Laboratory (PRF&L). Protocols used were approved by the Animal Care and Use Committee (IACUC) of PRF&L under numbers PRF2A and PRF2B. PRF&L is fully accredited by the Association for Assessment and Accreditation of Laboratory Animal Care (AAALAC) International and by the National Institutes of Health (NIH) Office of Laboratory Animal Welfare (OLAW.) The assurance number is A3886-01 with an expiration date of January 31, 2013.

### Drosophila Stocks

All fly stocks were maintained on standard yeast-agar media and kept at 18°C or 25°C. Flies used for screening were obtained from either Flytrap, the GFP protein trap database at Yale University (http://flytrap.med.yale.edu/), or from Dr. Allan C. Spradling. These fly lines contain the coding region of Green Fluorescent Protein (GFP) randomly inserted throughout the *Drosophila* genome. *Drosophila* proteins tagged with GFP are created by P element-mediated insertion into genes of an artificial exon encoding GFP flanked by splice acceptor and splice donor sites. Expression of GFP requires splicing into mature mRNAs and in-frame fusion [13]. Other fly stocks used in these studies include Oregon R *lamin C ^Ex296^* [39], *lamin^04643^* [40], *lamin^SZ18^* [41].

### Protein Trap Screening

Collections of GFP trap lines were screened for the nuclear distribution of the GFP signal; salivary glands and imaginal tissue from third instar larvae were dissected in 1 X Phosphate Buffered Saline (PBS) and mounted on clean slides using Vectashield containing the DNA stain DAPI to mark the nuclei. When mounting, the tissue was gently squashed or spread for improved visualization of individual cells. Adult ovaries from specific lines were dissected in a similar manner. Once prepared, samples were visualized using a Zeiss Axioplan fluorescence microscope and IP Lab software. Samples were screened to identify the cellular location of the GFP fluorescence. Those with strong nuclear localization, particularly those in which GFP was distributed in unique patterns, were selected for further analysis. The Flytrap database (http://flytrap.med.yale.edu/) was then used to obtain information on the location of the GFP insertion site; this information was confirmed using inverse PCR analysis of the PEP P-element as detailed by the Berkeley Drosophila Genome Project [42]. Out of this selected collection, the following protein trap lines have been studied more closely and will be the focus of the results described in this manuscript: CA06844 (CG11138), CA06872 (dNlp), CA07249 (Stwl) and G00196 (Df31).

### Immunolocalization Analysis

For analysis of imaginal tissues, third instar larvae were dissected in 100 mM sodium phosphate (pH 7.2) and fixed for 15 min with 4% paraformaldehyde in 100 mM sodium phosphate at room temperature [43]. Tissue was then rinsed 3 times for 10 min each in 100 mM sodium phosphate containing 0.1% Triton X-100 (sodium phosphate/TX). Tissue was then blocked using sodium phosphate/TX containing 5% normal goat serum for 30 min at room temperature. Appropriate dilutions of primary antibodies were done using blocking solution and incubated overnight at 4°C. Tissue was then washed three times in sodium phosphate/TX for 10 min each and the appropriate secondary antibodies diluted in blocking solution were added for 2 hr at room temperature. The washes were repeated as described above and samples were then stained with 1 X DAPI (0.05 µg/ml) for 2–4 min. Samples were rinsed in 100 mM phosphate buffer and then mounted using Vectashield anti-fade mounting medium.

Immunostaining of Kc cells was done as described above. For immunostaining of polytene chromosomes, third instar larvae were dissected in 0.7% NaCl and the salivary glands were removed [44]. Each salivary gland pair was placed in 4% paraformaldehyde, 1 X PBS, 0.1% TX (PBT) for 2 min at room temperature. The tissue was then incubated in 45% acetic acid for 5 min before being placed in a drop of 45% acetic acid on a 0.1% poly-L-lysine coated slide. After squashing, slides were incubated for 30 min in blocking solution with 5% bovine serum albumin in PBS. Primary antibodies diluted in 5% normal goat serum in PBT were then added and incubated overnight at 4°C. Slides were washed three times for 10 min each using PBT and one 5 min wash in PBS. Secondary antibodies were then added diluted in 5% normal goat serum in PBT and incubated 2 hr at room temperature. Washes were then repeated as above and slides were stained in 1X DAPI for 2–4 min and rinsed with PBS. Slides were then mounted using Vectashield anti-fade mounting medium. When using chromosomes from heat shocked larvae, third instar larvae were suspended in an Eppendorf tube in a 37°C water bath for 20 min and then dissected immediately in 0.7% NaCl. Polytene chromosomes were then fixed and immunostained as described [45].

Immunostaining of adult ovaries was done as described [46]. Following incubation of primary antibodies, samples were washed twice for 10 minutes in 3 mM NaH_2_PO_4_, 7 mM Na_2_HPO_4_, 130 mM NaCl, and.125% Tween 20 (PBT) and once for 10 min in PBT plus 1.5% BSA (PBTA) again using solutions described in McKearin and Ohlstein. Samples were then incubated in secondary antibodies in PBTA for 2 hr at room temperature and then washed three times for 10 min each in PBT [46]. Ovaries were lastly stained with 1×DAPI for 8–10 minutes and then mounted using vectashield antifade.

The following primary antibodies were used: rabbit α-GFP (Molecular Probes) 1∶400, rat α-Stwl (gift from Dr. Dennis McKearin) 1∶20, guinea pig α-Stwl 1∶800, rabbit α-CG11138 1∶1000, rabbit α-Df31 1∶400, rabbit α-dNlp (gift from Dr. James Kadonaga) 1∶200, mouse α-Lamin Dm0 (Developmental Studies Hybridoma Bank) 1∶200, mouse α-Lamin C (Developmental Studies Hybridoma Bank) 1∶200, rabbit α-CP190 1∶4000, rat α-Vasa (Developmental Studies Hybridoma Bank) 1∶200, rat α-Mod(mdg4)2.2 1∶1000, rabbit α-Su(Hw) 1∶2000, mouse α-MSL1 (gift from Dr. John Lucchesi), rabbit α-histone H3S10 ph (Millipore), rabbit α- phospho-Pol IIser5 (H14, Covance) and guinea pig α-CTCF 1∶2000. Antibodies complexed to Alexafluor 488 and Alexafluor 594 targeting the appropriate primary antibodies were used as secondary antibodies.

### Preparation of Antibodies

For preparation of antibodies against the CG11138, Stwl and Df31 proteins, fragments from each protein containing N-terminal GST-tags and C-terminal His-tags were purified from *Escherichia coli* on nickel-agarose columns (Quiagen) and were used to immunize rabbits using standard procedures. Protein fragments include amino acids 358–615 for CG11138, 600–1025 for Stwl and 1–185 for Df31. The specificity of the resulting antibodies was validated by western analysis and immunostaining of polytene chromosomes and imaginal tissue from wild type and mutant larvae. In all three cases, antibodies gave the same results as antibodies to GFP used in similar analyses with strains carrying GFP protein traps in each of the genes.

### Western Analysis

Extracts from third instar larva imaginal tissue and Kc cells were prepared using standard protocols and run on tris-glycine-SDS gels [44]. The Millipore SNAP i.d. detection system was used for immunodetection and Thermo Scientific chemiluminescent substrates were used for visualization as per manufacturer’s specifications. The following primary antibodies were used: rabbit α-GFP (1∶3000), rabbit α-CG11138 (1∶3000), guinea pig α-Stwl (1∶5000), rabbit α-Df31 (1∶5000), mouse α-Lamin C (1∶2000) and all appropriate HRP-conjugated secondary antibodies were used at 1∶3000 dilution.

## Supporting Information

Figure S1 **GFP autofluorescence in protein-trap alleles depicting both nuclear and nonnuclear GFP localization.** (A) Protein-trap allele CA07367 showing nonnuclear GFP localization in diploid cells. (B) Protein-trap allele CC006263 with nonnuclear GFP in salivary gland nuclei containing polytene chromosomes. (C) Df31 protein-trap allele G00196 with nuclear GFP in diploid cells showing distinct foci of localization amidst more diffuse GFP fluorescence. (D) Salivary gland nuclei of the Df31 protein-trap allele G00196 showing GFP fluorescence predominantly surrounding the polytene chromosomes. In all panels DNA is labeled blue using DAPI and the GFP is green.
(TIF)

Figure S2 **Western blot analysis of CG11138 protein.** Lanes 1, 2, and 3 contain increasing amounts of extract from wild type OR third instar larvae imaginal tissue. Lanes 4, 5 and 6 contain increasing amounts of extract from CG11138 protein-trap allele third instar larvae imaginal tissue. Lanes 7, 8 and 9 contain increasing amounts of extract from Df31 protein-trap allele third instar larvae imaginal tissue. As can be seen in lanes 1, 2 and 3 the predominant isoform of CG11138 has a molecular weight of ca. 65 kDa, similar to that predicted for isoforms CG11138-PC and CG11138-PD. In addition, there are two larger bands of about 100 kDa and 150 kDa, respectively. The nature of these bands is unclear but they appear to be specific for CG11138, as their size shifts in rows 4, 5 and 6, which contain the CG11138-GFP fusion protein. Lanes 7, 8 and 9 suggest that this shift is specific for CG11138-GFP, as flies expressing a Df31-GFP protein show bands matching the wild type OR extracts when probed for CG11138.
(TIF)

Table S1 Summary of protein-trap screen results.
(XLSX)
