# Supplementary figures and images for: Identification and Characterization of Proteins Involved in Nuclear Organization Using Drosophila GFP Protein Trap Lines
Source: PLoS One. 2013 Jan 16;8(1):e53091. doi: 10.1371/journal.pone.0053091 (PMC3547006; doi:10.1371/journal.pone.0053091)

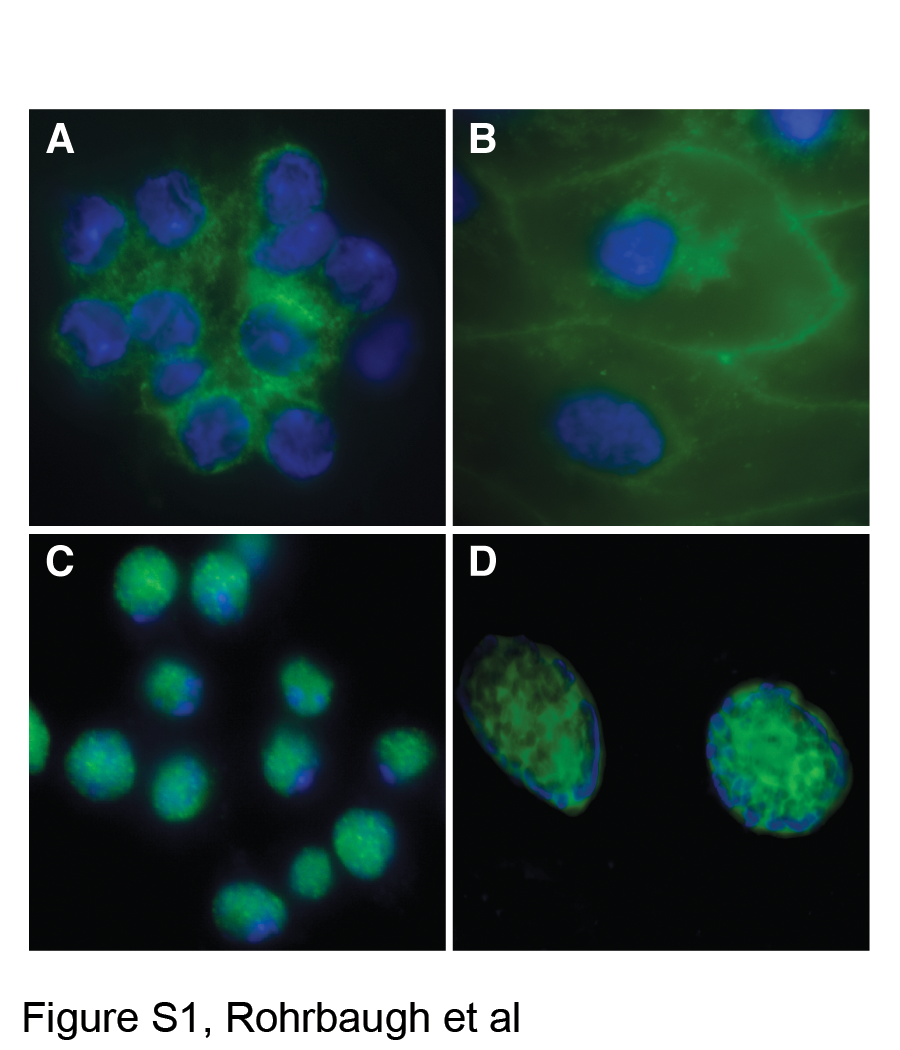

Supplement: Figure S1 — GFP autofluorescence in protein-trap alleles depicting both nuclear and nonnuclear GFP localization. (A) Protein-trap allele CA07367 showing nonnuclear GFP localization in diploid cells. (B) Protein-trap allele CC006263 with nonnuclear GFP in salivary gland nuclei containing polytene chromosomes. (C) Df31 protein-trap allele G00196 with nuclear GFP in diploid cells showing distinct foci of localization amidst more diffuse GFP fluorescence. (D) Salivary gland nuclei of the Df31 protein-trap allele G00196 showing GFP fluorescence predominantly surrounding the polytene chromosomes. In all panels DNA is labeled blue using DAPI and the GFP is green. (TIF) [file pone.0053091.s001.tif]

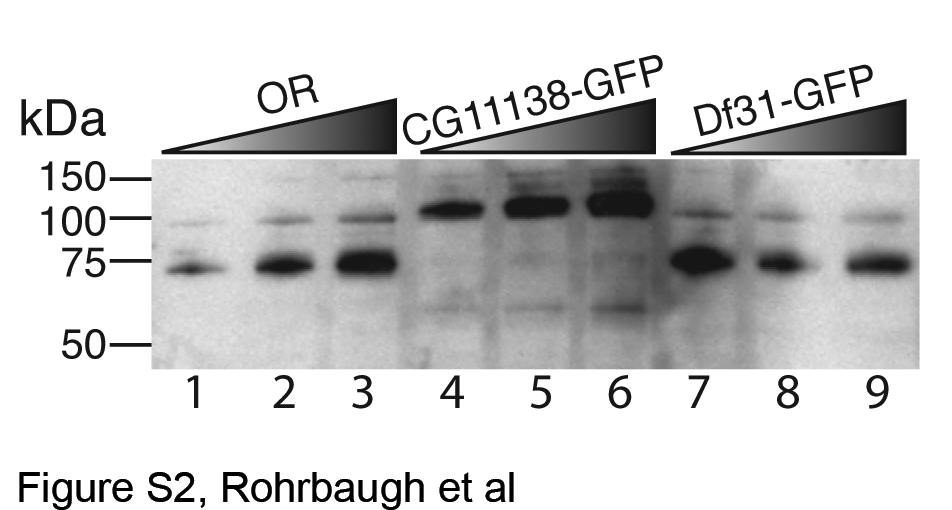

Supplement: Figure S2 — Western blot analysis of CG11138 protein. Lanes 1, 2, and 3 contain increasing amounts of extract from wild type OR third instar larvae imaginal tissue. Lanes 4, 5 and 6 contain increasing amounts of extract from CG11138 protein-trap allele third instar larvae imaginal tissue. Lanes 7, 8 and 9 contain increasing amounts of extract from Df31 protein-trap allele third instar larvae imaginal tissue. As can be seen in lanes 1, 2 and 3 the predominant isoform of CG11138 has a molecular weight of ca. 65 kDa, similar to that predicted for isoforms CG11138-PC and CG11138-PD. In addition, there are two larger bands of about 100 kDa and 150 kDa, respectively. The nature of these bands is unclear but they appear to be specific for CG11138, as their size shifts in rows 4, 5 and 6, which contain the CG11138-GFP fusion protein. Lanes 7, 8 and 9 suggest that this shift is specific for CG11138-GFP, as flies expressing a Df31-GFP protein show bands matching the wild type OR extracts when probed for CG11138. (TIF) [file pone.0053091.s002.tif]
